# Bouveret's Syndrome Presenting as Jejunal Obstruction: A Case Report

**DOI:** 10.7759/cureus.77617

**Published:** 2025-01-18

**Authors:** Mariana Sousa, Madalena Santos, João Francisco Abrantes, Lígia Peixoto

**Affiliations:** 1 Internal Medicine, Unidade Local de Saúde Santa Maria - Hospital de Santa Maria, Lisbon, PRT

**Keywords:** bouveret's syndrome, cholecystoduodenal fistula, gallstone, gallstone ileus, pneumobilia

## Abstract

Bouveret's syndrome, a rare and severe complication of gallstone disease, is characterized by gastric outlet obstruction resulting from the passage of a gallstone through a bilioenteric fistula, typically a cholecystoduodenal fistula. We present the case of a 68-year-old female patient with bilateral low back pain, nausea, vomiting, and constipation. Imaging revealed jejunal obstruction due to gallstone migration through a cholecystoduodenal fistula. Initial management involved nasogastric decompression and fluid resuscitation, followed by enterolithotomy. Diagnosis of Bouveret's syndrome is challenging due to nonspecific symptoms. Imaging, particularly computed tomography (CT), is essential for identifying Rigler's triad: pneumobilia, bowel obstruction, and ectopic gallstone. Early intervention is essential to avoid complications. This case highlights the complexity of diagnosing and managing Bouveret's syndrome. A high degree of clinical suspicion is essential for accurate diagnosis and timely treatment, ensuring the optimal management of Bouveret's syndrome.

## Introduction

Bouveret's syndrome, a rare and severe complication of gallstone disease, is characterized by gastric outlet obstruction resulting from the passage of a gallstone through a bilioenteric fistula, typically a cholecystoduodenal fistula. The chronic inflammation from gallstone disease leads to fistula formation and the subsequent migration of stones into the gastrointestinal tract. Beaussier described Bouveret's syndrome for the first time in 1770. However, Leon August Bouveret was the first to report two cases in 1896 [1].

The prevalence of gallstone ileus represents 0.3-0.5% of patients with gallstones, and Bouveret's syndrome occurs only in 1-3% of ileus [2,3]. In this report, we discuss a case of Bouveret's syndrome, emphasizing its clinical presentation and management strategies.

## Case presentation

A 68-year-old female patient with a history of type 2 diabetes, hypertension, dyslipidemia, and chronic kidney disease KDIGO 3b with a single kidney presented to the emergency department due to complaints of bilateral low back pain that radiated to the abdomen, nausea and persistent vomiting for the last six days, worsening the day before, and constipation for the last four days.

On physical examination, she was conscious and hypotensive, with pain on abdominal palpation of the left quadrants and positive renal murphy on the left.

Table 1 reveals the main laboratory tests.

**Table 1:** Laboratory findings of the patient at admission.

Laboratory parameters	Value (units)	Reference value
Hemoglobin	14.9 g/dL	12.0-15.3
Leukocytes	32.3×10^9^/L	4-0-11.0
Neutrophils	30.7×10^9^/L	1.9-7.5
C-reactive protein	11.9 mg/L	<0.5
Procalcitonin	4.33 ng/mL	<0.5
Urea	280 mg/dL	16-49
Creatinine	8.37 mg/dL	0.5-0.9
Aspartate aminotransferase	13 U/L	0-32
Alanine aminotransferase	<6 U/L	0-33
Gamma-glutamyl transferase	19 U/L	0-40
Total bilirubin	0.61 mg/dL	<1.2

Renal ultrasound did not show dilation of the excretory tree of the single left kidney nor free fluid or collections in perirenal topography.

**Figure 1:**
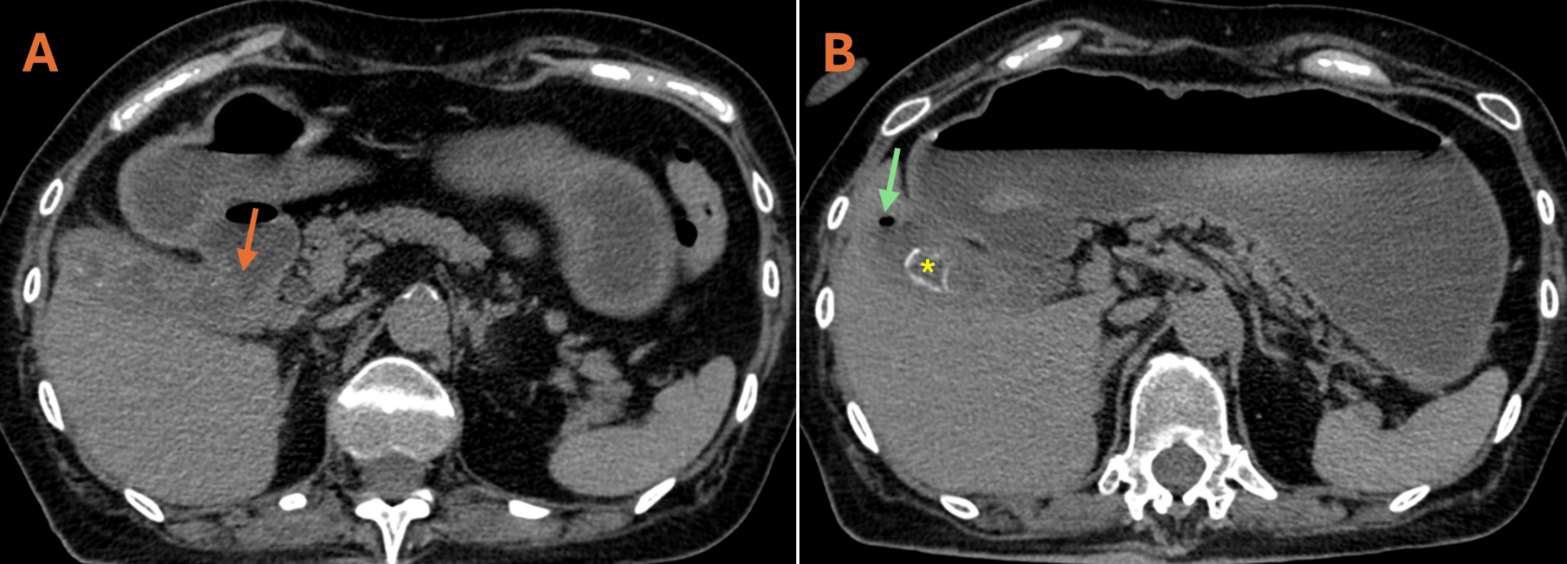
Cholecystoduodenal fistula. Axial CT slices without contrast. In image A, there is a lack of definition of the anterior wall of the gallbladder body and the first portion of the duodenum, suggesting a cholecystoduodenal fistula (orange arrow). In image B, the presence of mineralized calculus (green arrow) and gas (asterisk) in the gallbladder lumen can be identified. CT: computed tomography

**Figure 2:**
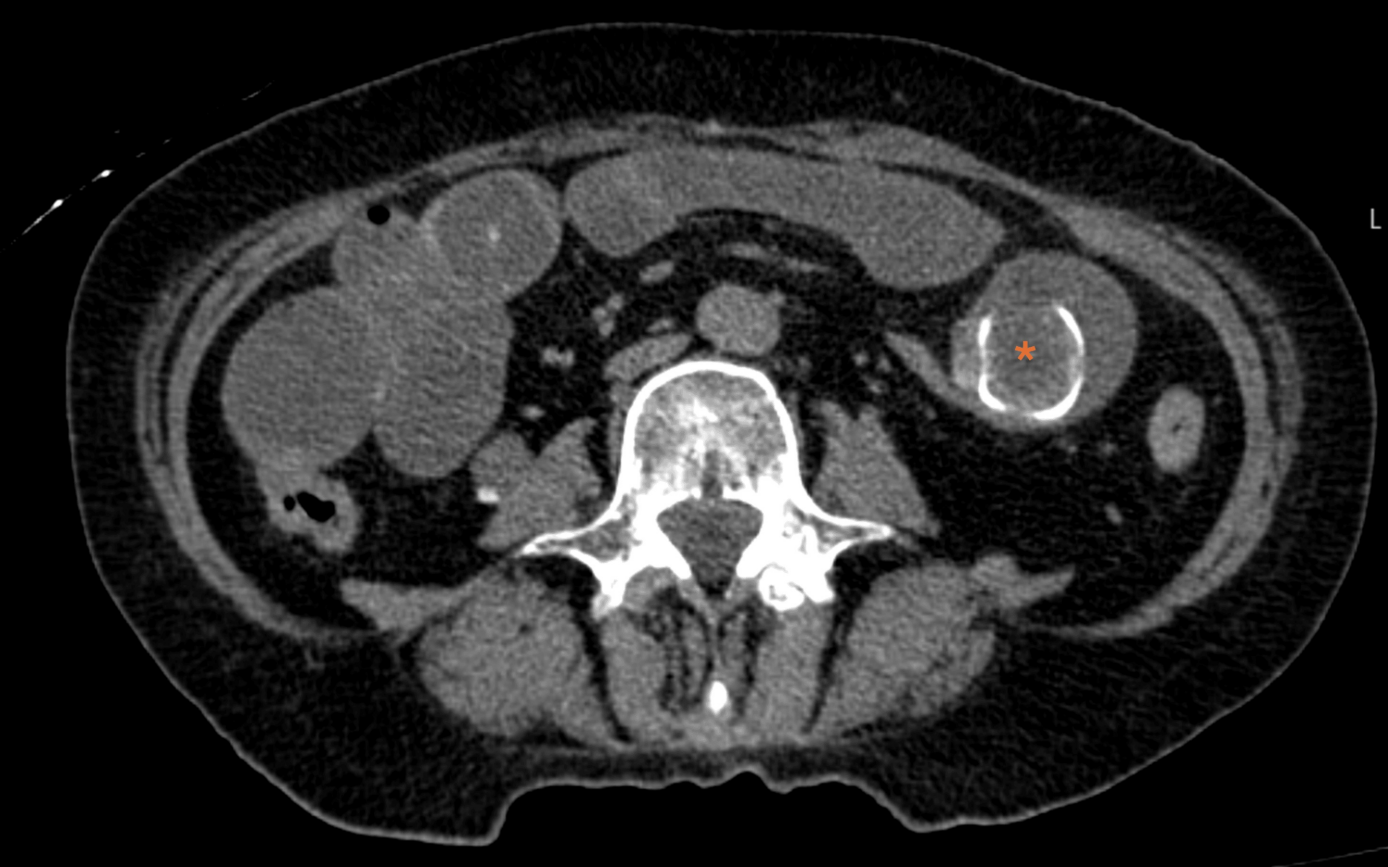
Non-contrast CT showing the migration of a mineralized biliary stone (asterisk) into the lumen of a jejunal loop. CT: computed tomography

Computed tomography (CT) revealed signs of jejunal obstruction due to an oval formation with multilayer mineralization at the periphery measuring 38 mm; another spheroid-like structure measuring approximately 26 mm in the lumen of the upstream jejunum; gallbladder with two stones and gas inside, as well as in the cystic duct and bile ducts of the left lobe, assuming origin from the gallbladder; and a vague and discrete thickening of the anterior wall of the vesicular body and the first portion of the duodenum, changes that in the current clinical context could suggest a cholecystoduodenal fistula with migration of larger stones found in the jejunal lumen (Figure 1 and Figure 2). These changes were compatible with biliary ileus.

The nasogastric tube was placed with drainage of fecaloid contents. Shock progressed with oliguria, undergoing volume resuscitation (3L) and starting antibiotic therapy with ceftriaxone. The patient underwent enterolithotomy with enterorrhaphy. There were no complications in the perioperative period.

## Discussion

This case illustrates a rare but significant complication of gallstone disease.

Risk factors for Bouveret's syndrome include a history of cholelithiasis, gallstones >2.5 cm in size, the female gender, and age >70 years. It has been reported that approximately 43-68% of patients have a history of recurrent biliary colic, jaundice, or acute cholecystitis [4].

The clinical presentation of Bouveret's syndrome can be nonspecific and depends on various factors, like the impaction site of the stone and the degree of the obstruction. It often includes nausea, vomiting, and abdominal pain, which are nonspecific and can delay diagnosis. In this patient, the persistent vomiting and abdominal pain, along with her background of constipation, were suggestive of gastrointestinal obstruction. The diagnostic challenge was heightened by her comorbidities, particularly chronic kidney disease, which required the careful consideration of therapeutic strategies.

Laboratory findings are typically nonspecific and may show elevation of hepatic cytocholestasis markers in about one-third of the patients. In this case, the evaluation revealed systemic inflammatory response syndrome (SIRS), as evidenced by leukocytosis and elevated C-reactive protein and procalcitonin levels, albeit with normal liver function [5,6].

The constellation of pneumobilia, bowel obstruction, and an aberrant gallstone referred to as Rigler's triad is highly suggestive of Bouveret's syndrome but is only found in 40-50% of cases. Imaging was pivotal in establishing the diagnosis. The CT findings of a cholecystoduodenal fistula, pneumobilia, and the presence of gallstones in the jejunal lumen confirmed the diagnosis of gallstone ileus, a rare but recognized complication of Bouveret's syndrome. While ultrasound is a common initial imaging modality, it may fail to detect the fistula or the ectopic gallstones, underscoring the value of advanced cross-sectional imaging in complex cases [5-7].

The treatment of Bouveret's syndrome involves addressing both the obstruction and the underlying gallstone disease. In this case, nasogastric decompression provided initial symptom relief by reducing gastric distension and relieving the obstruction proximally.

The optimal treatment strategy for Bouveret's syndrome remains controversial and is dependent on the patient's condition, the location of the obstruction, the size of the stone and fistula, and the presence of residual gallstone. In general, endoscopic retrieval or minimally invasive lithotripsy should be attempted initially. However, in critically ill patients, a surgical approach aligns with the principle of damage control surgery, prioritizing the immediate resolution of life-threatening obstruction over definitive fistula repair. Subsequent management of the fistula can be deferred to a later stage, depending on the patient's recovery and overall health status. In this case, enterolithotomy was performed to remove the obstructing stones, a procedure associated with relatively low morbidity compared to more extensive surgeries like fistula repair [6,8].

Early intervention and appropriate surgical management are crucial in Bouveret's syndrome to prevent complications such as perforation, sepsis, and worsening multiorgan dysfunction. Despite her critical presentation, the patient's outcome was favorable, likely due to prompt diagnosis, early resuscitation, and timely surgical intervention. However, her pre-existing comorbidities, particularly chronic kidney disease, underscore the need for long-term monitoring and comprehensive follow-up care to prevent recurrent biliary events and assess for the delayed complications of the bilioenteric fistula.

## Conclusions

This case highlights the complexity of diagnosing and managing Bouveret's syndrome, particularly in patients with complex medical histories. A high degree of clinical suspicion is essential for accurate diagnosis and timely treatment, ensuring the optimal management of Bouveret's syndrome.

Given the rarity of Bouveret's syndrome, detailed case reports and discussions such as this contribute to the growing body of literature and enhance the understanding of this challenging condition.
